# Resveratrol pretreatment mitigates LPS-induced acute lung injury by regulating conventional dendritic cells’ maturation and function

**DOI:** 10.1515/biol-2021-0110

**Published:** 2021-09-29

**Authors:** Bingnan Guo, Yigen Peng, Yuting Gu, Yi Zhong, Chenglei Su, Lin Liu, Dafei Chai, Tengfei Song, Ningjun Zhao, Xianliang Yan, Tie Xu

**Affiliations:** Jiangsu Institute of Health Emergency, Xuzhou Medical University, Xuzhou, Jiangsu 221004, China; Department of Emergency Medicine, The Affiliated Hospital of Xuzhou Medical University, Xuzhou, Jiangsu 221000, China; Department of Emergency Medicine, The Affiliated Jiangning Hospital of Nanjing Medical University, Nanjing, Jiangsu 211100, China; Cancer Institute, Xuzhou Medical University, Xuzhou, Jiangsu 221004, China; The Feinstein Institute for Medical Research, Manhasset, NY 11030, New York, United States

**Keywords:** resveratrol, LPS, acute lung injury, conventional dendritic cells

## Abstract

Acute lung injury (ALI)/acute respiratory distress syndrome (ARDS) is a severe syndrome lacking efficient therapy and resulting in high morbidity and mortality. Although resveratrol (RES), a natural phytoalexin, has been reported to protect the ALI by suppressing the inflammatory response, the detailed mechanism of how RES affected the immune system is poorly studied. Pulmonary conventional dendritic cells (cDCs) are critically involved in the pathogenesis of inflammatory lung diseases including ALI. In this study, we aimed to investigate the protective role of RES via pulmonary cDCs in lipopolysaccharide (LPS)-induced ALI mice. Murine ALI model was established by intratracheally challenging with 5 mg/kg LPS. We found that RES pretreatment could mitigate LPS-induced ALI. Additionally, proinflammatory-skewed cytokines decreased whereas anti-inflammatory-related cytokines increased in bronchoalveolar lavage fluid by RES pretreatment. Mechanistically, RES regulated pulmonary cDCs’ maturation and function, exhibiting lower level of CD80, CD86, major histocompatibility complex (MHC) II expression, and IL-10 secretion in ALI mice. Furthermore, RES modulated the balance between proinflammation and anti-inflammation of cDCs. Moreover, *in vitro* RES pretreatment regulated the maturation and function of bone marrow derived dendritic cells (BMDCs). Finally, the adoptive transfer of RES-pretreated BMDCs enhanced recovery of ALI. Thus, these data might further extend our understanding of a protective role of RES in regulating pulmonary cDCs against ALI.

## 1 Introduction

Acute lung injury (ALI) or acute respiratory distress syndrome (ARDS) is defined as an inflammation-mediated severe pulmonary disease in the emergency intensive care unit with high mortality and morbidity [1,2]. Globally, over one-third of ARDS patients failed to respond to treatment, eventually leading to death [3]. As a life-threatening critical-care illness, sepsis and endotoxemia are major triggers of ALI [4] featured by cytokine-mediated excessive inflammation and inflammatory dysfunction [5]. Therefore, physiologically, inflammatory dysfunction is the crucial mechanism underlying ALI [6]. In a mouse model, induction of ALI with intratracheal administration of lipopolysaccharide (LPS) [7], an outer membrane of gram-negative bacteria glycolipid, is extensively used to simulate sepsis or endotoxemia-mediated ALI patients. Despite research progress over decades in the field of ALI, little effective therapeutic approaches or agents are available against ALI. Thus, how therapeutic agents limit inflammation is a critical process for controlling ALI development.

As a functional and phenotypic heterogeneity of antigen-presenting cells (APCs), dendritic cells (DCs), including conventional dendritic cells (cDCs) and plasmacytoid DCs (pDCs), are the frontline for the priming phase of immune response during inflammation and infection [8,9]. Moreover, the application of DC-based therapy was widely used for various diseases [10,11] and several reports have proven the possibility of mitigating ALI by modulating DCs [12–14]. Mature or activated DCs express high level of co-stimulatory molecules, including CD80, CD86 and MHC II [9]. Binding these ligands to receptors is essential for T cell activation [15]. In response to exogenous agents, cDCs in peripheral tissues capture pathogens and undergo maturation, followed by distribution to lymph nodes or inflammation and infection sites, including mucosal tissues [16]. Thereby, cDCs are believed to have a great capacity to prime Th cells, resulting in regulating Th1, Th2, and Th17 responses. Previous reports have indicated that cytokines related to Th1 and Th17 were driven by the maturation and accumulation of cDCs [17]. Conversely, Th2-related cytokines were associated with the obstruction of cDC maturation and increment [18]. Several studies have reported that pulmonary cDCs are involved in a various pulmonary inflammation diseases, such as allergy, respiratory viral infection, and inflammation-mediated lung injury [19–21]. Furthermore, regulating pulmonary cDCs has shown promising therapeutic outcome in a murine ALI model [13]. Thus, governing and modulating DC maturation and function may greatly benefit ALI treatment.

Resveratrol (3,4,5-trihydroxy-trans-stilbene; RES) is a natural polyphenol produced by plants, typically mulberries, peanuts, and red grapes [22]. RES has multiple anti-inflammatory properties through the inhibition of macrophage activation in brain [23], suppression of CD4 T cell activation in rheumatoid arthritis [24], or limitation of NLRP3 inflammasome in hepatic metaflammation [25]. Meanwhile, other studies have shown that RES could mitigate lung inflammation via various mechanisms in mouse models [26,27]. Simultaneously, in ALI/ARDS, cDCs are considered to be pro‑inflammatory initiator and mediator that deteriorate ALI at the early stage [14]. Thus, we hypothesized that RES pretreatment could modulate cDCs maturation and function to decrease excessive inflammation and thus, alleviate LPS-induced ALI.

In summary, the present study aimed to determine whether RES could attenuate LPS-induced ALI by regulating cDC maturation and function *in vitro* and *in vivo*. First, we confirmed that RES could significantly alleviate LPS-induced ALI in a murine ALI model. Moreover, RES pretreatment reduced the Th1-skewed cytokines, while increased the Th2-skewed cytokines expression in bronchoalveolar lavage fluid (BALF). Mechanistically, RES could regulate pulmonary cDC maturation and function, exhibiting lower level of CD80, CD86, MHC II expression, and IL-10 secretion in LPS-induced ALI mice. RES modulated the balance between proinflammation and anti-inflammation of cDCs. RES pretreatment also regulated the maturation and function of bone marrow derived dendritic cells (BMDCs) *in vitro*. Finally, the adoptive transfer of RES-pretreated BMDCs enhanced the recovery of ALI. Thus, our study indicated that RES participated in protecting against the pathological process of ALI by modulating the maturation and function of cDCs.

## 2 Materials and methods

### 2.1 Animals and agents

Six- to eight- weeks-old female C57BL/6 (H-2^b^) mice were purchased from experimental animal center of Chinese Academy of Science. All mice were bred and maintained under pathogen-free conditions. All mice were group housed under a 12 h light/dark cycle with ad libitum access to food and water. All mice were euthanized via carbon dioxide inhalation. RES was purchased from Sigma (R5010, USA) and dissolved in DMSO.

**Ethical approval:** The research related to animal use has been complied with all the relevant national regulations and institutional policies for the care and use of animals and was approved by the Care and Use of Laboratory Animals (Ministry of Health, China, 1998) and the guidelines of the Laboratory Animal Ethical Committee of Xuzhou Medical University (Assurances No. L20190226366).

### 2.2 Mouse model of LPS-induced ALI

The general procedure modeling LPS-induced ALI has been described in previous study [12]. Briefly, mice were anesthetized and placed in a supine position upon a board with head tilted at 37°C. LPS (055:B5, Sigma, USA) was dissolved in 0.9% of saline and intratracheally injected to the trachea at a dosage of 5 mg/kg [28] with a 22-gauge feeding needle. Then, the mice were placed in a vertical position and rotated for 30 s to distribute the LPS evenly within the lungs. Mice in the control group received the same volume of 0.9% of saline and manipulations. All mice were euthanized at 24 h after LPS or saline administration. For mice survival assay, mice survival was monitored daily up to ten days post-challenge.

### 2.3 Drug treatment

**Figure 1:**
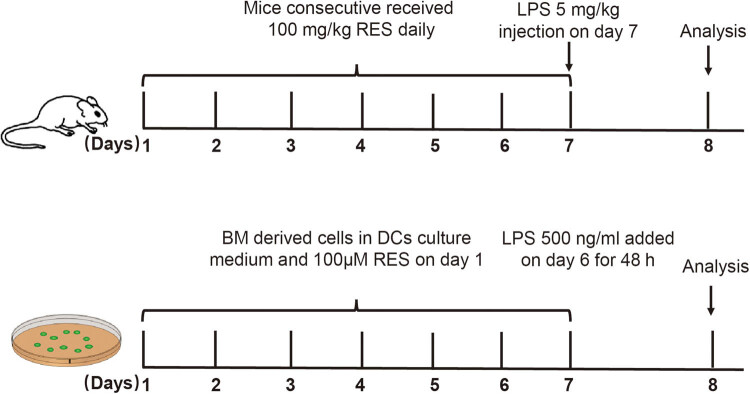
Schematic diagram of experimental protocol.

For *in vivo* assay, RES was administered orally using a 30 mm oral gavage needle at 100 mg/kg in a total volume of 100 μL in an appropriate vehicle of 1% of carboxymethyl cellulose (CMC), a dose established in previous studies [29,30]. RES was given daily for seven days prior to LPS administration for ALI model. For *in vitro* assay, BMDCs were generated as described below in cell culture section. 0–100 μM of RES was added to the culture medium on initial day, and 500 ng/mL of LPS (055:B5, Sigma, USA) was added to the culture medium on day six. 48 h later, BMDCs were harvested for future experiments [31]. In some experiments, methylthiazolyldiphenyl-tetrazolium bromide (MTT) (C0009S, Beyotime, China) assay was performed to evaluate the viability and cytotoxicity of RES pretreated BMDCs. The schematic diagram of the experimental protocol is shown in Figure 1.

### 2.4 Histologic examination

Lung tissues from the mice were removed after 24 h of LPS administration and were fixed in 10% of phosphate-buffered formalin, paraffin-embedded, sectioned, and stained with hematoxylin and eosin (HE), and examined by optical microscope (DM3000b, Leica, Germany) with 200× magnifications. The histopathological changes in mice lung injury were compared quantitatively by calculating the histopathological scores [32], which were scored based on the assignment of one point for each of the following parameters: 1 = Alveolar hyperemia, 2 = Hemorrhage, 3 = Interstitial or leukocyte infiltration or aggregation, and 4 = Alveolar septal thickening. Two independent researchers scored separately in a blinded manner.

### 2.5 Lung wet/dry (W/D) weight ratios

The middle lobe of right lung was excised and the wet weight was recorded. The lung was then placed in an incubator at 80°C for at least 72 h to obtain the dry weight. The ratio of wet lung to dry lung was calculated.

### 2.6 MPO activity

Briefly, lung tissue samples were thawed and homogenized in myeloperoxidase (MPO) assay buffer (Abcam). The samples were then freeze-thawed and centrifuged at 12,000*g* for 10 min at 4°C. The enzyme activity was tested by MPO activity Kit (ab105136, Abcam, UK) according to manufacturer’s instructions using a microplate reader (Biotek, USA). Results were expressed as MPO units per gram of tissue.

### 2.7 Cytokines measurement

The BALF was collected at indicated time by using three consecutive instillations of 1 mL of PBS at room temperature. The collected BALF of 1,500*g* was centrifuged at 4°C for 5 min, and the supernatants were stored at −80°C for estimation of cytokine levels. proinflammatory (Th1)- and anti-inflammatory (Th2)-related cytokines [33] were measured by ELISA (BioLegend, USA) kits according to the manufacturers’ instructions.

### 2.8 Isolation of pulmonary hematopoietic cells

The pulmonary hematopoietic cells’ isolation procedure has been described in our previous study [34]. Briefly, lungs were harvested after perfusion with 10 mL of cold PBS. Tissues were minced and digested in Hank’s buffer containing Liberase TM (05401127001, Roche, Switzerland) and DNase I (B00003, Roche, Switzerland). Single cell suspension was collected by filtering through a 70 μm cell strainer. In some experiments, lymphocytes were further purified using 40% of Percoll gradient centrifugation.

### 2.9 Flow cytometry and cell sorting

Antibodies (Abs) were purchased from BioLegend (USA) or eBioscience (USA). Abs used included anti-CD45(A20), anti-CD4(RM4-5), anti-CD11b(M1/70), anti-CD11c(N418), anti-F4/80(BM8), anti-CD25(3C7), anti-CD80(16-10A1), anti-CD86(GL-1), anti-MHCII (M5/114.15.2), anti-ILT3(H1.1), anti-IL10(JES5-16E3), anti-IL12(C15.6), anti-IL17(9B10), anti-Foxp3(FJK-16s), and isotype control antibody. For DCs staining, prepared lung mononuclear cells (MNCs) and BMDCs were staining F4/80 first, then F4/80^−^ cells were further stained with CD11b and CD11c, since DCs express the marker CD11c, but not the marker F4/80 on the cell surface [14]. Intracellular staining of transcription factors was performed using Foxp3 Fix/Perm Kit (Thermo, USA) according to the manufacturer’s instructions. Flow cytometric analysis was performed on FACSCanto II (BD Bioscience, USA). Cell sorting was performed using an FACSAria III (BD Biosciences, USA).

### 2.10 Cell culture

BMDCs were generated as described previously [35]. Briefly, murine bone marrow from the femura and tibiae of C57BL/6 mice were flushed and depleted of red blood cells (RBC) by ACK lysis buffer (Sigma, USA). Cells (1 × 10^6^ cells/mL) were grown in 100 mm dishes in RPMI 1640 containing 10% of FBS, l-glutamine, nonessential amino acids, sodium pyruvate, penicillin–streptomycin, HEPES, and 2-ME (Sigma, USA). rmGM-CSF (40 ng/mL, AF31503, PeproTech, USA), rmIL-4 (40 ng/mL, 21414, PeproTech, USA), and 0–100 μM RES were added to the culture medium in a humidified 5% CO_2_ incubator at 37°C on day 0; on day six, non-adherent cells were washed out, adherent cell clusters were harvested, and purified with anti-CD11c beads (Miltenyi Biotec, Germany). Subsequently, 500 ng/mL of LPS was added to the purified cells for 48 h. On day eight, cells were collected for analysis or future experiments. For RES-treated BMDCs co-cultured with sorted CD4 from ALI mice, two kinds of cells (ratio 1:1 or 3:1) were co-cultured in the medium containing rmIL-2 (5 ng/mL, 21212, PeproTech, USA), rhTGF-β (5 ng/mL, 10021 C, PeproTech, USA), and rmIL-6 (20 ng/mL, 21616, PeproTech, USA) for three days and then the cells were stained with antibodies against CD25, Foxp3, IL-17, or IL-10 using intracellular or surface staining following flow cytometry analysis. In some experiments, sILT3 (10 μg/mL, 328978, ACROBiosystems, USA) were added into the co-cultured medium following flow cytometry analysis.

### 2.11 Adoptive cell transfer

C57BL/6 (H-2^b^) mice were immediately injected with 1 × 10^6^ RES-pretreated BMDCs via tail veil 1 day before LPS challenge and then the mice were euthanated after 24 h for future experiments.

### 2.12 Statistical analysis

Statistical analysis were performed using GraphPad Prism 6 Software (La Jolla, CA). Data were presented as mean values ± SEM and statistically analyzed with two-tailed independent Student’s *t*-test (two groups). For multiple groups, 1-way ANOVA was used for each experiment. For *in vivo* experiments, groups of 5–10 mice were used per experimental group. For *in vitro* assays, all experiments were performed in triplicate and one representative experiment was presented. For mice survival, Kaplan–Meyer analysis with log-rank test was used. The level of statistical significance was set at *p < 0.05, **p < 0.01, and ***p < 0.001.

## 3 Results

### 3.1 RES alleviated LPS-induced ALI

**Figure 2:**
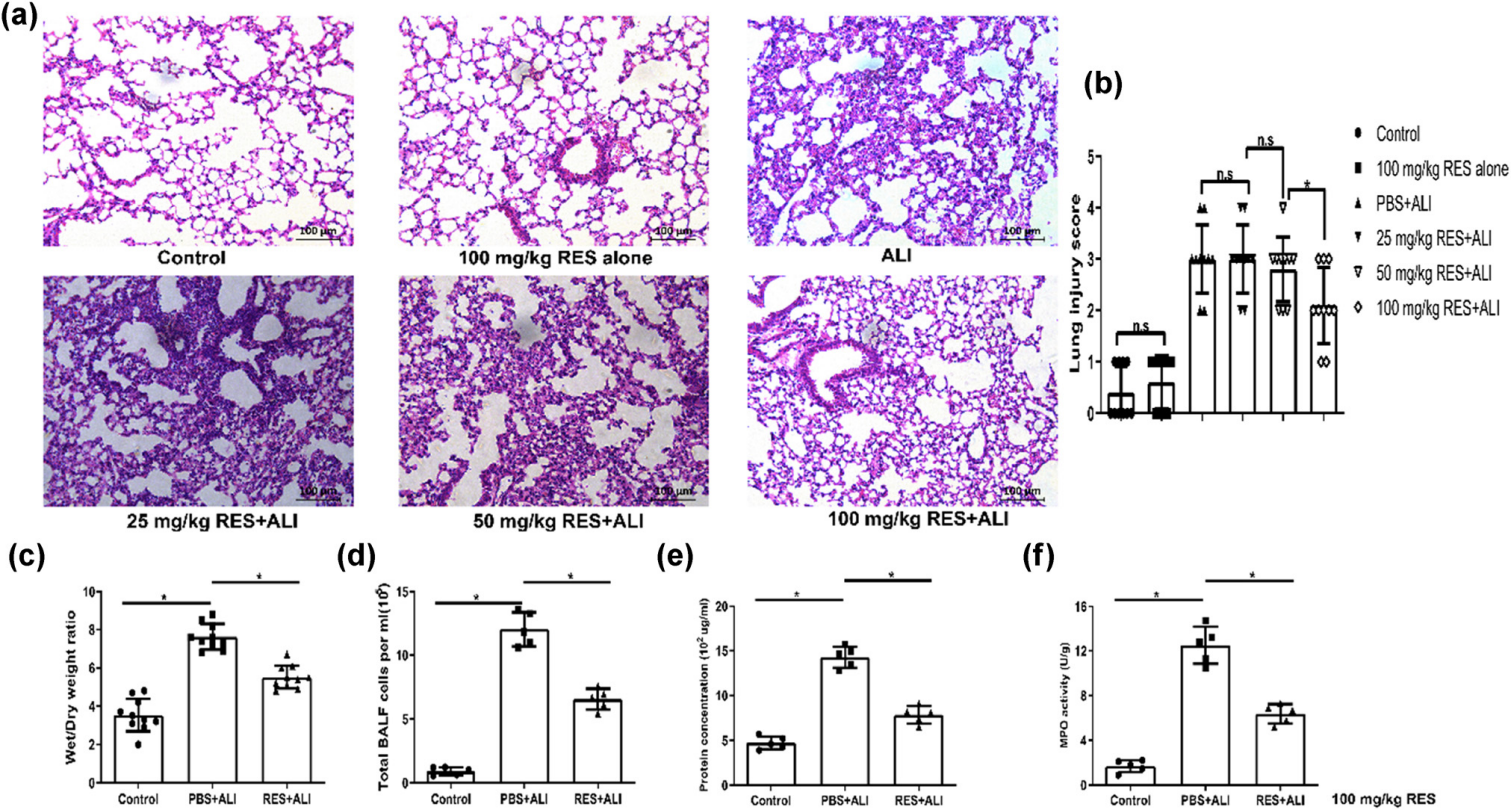
The alleviation of LPS-induced ALI by oral pretreatment with RES. RES was administrated orally daily for seven days prior to LPS administration for ALI model. Lung was harvested 24 h after LPS intratracheal administration. (a) The representative pulmonary HE section is shown for each group (magnification:200×); (b) pulmonary histopathological scores are determined in six groups at 24 h; (c) the wet lung-to-dry weight ratio (W/D ratio) in three groups at 24 h; (d and e) total cells and proteins in BALF were analyzed in three groups at 24 h; (f) lung MPO activity is calculated in three groups at 24 h. Data are from one representative experiment of three performed and presented as the mean value ± SEM, 1-way ANOVA was used for pulmonary histopathological scores and W/D ratio, *n* = 10; for cells and proteins in BALF and MPO activity, *n* = 5, *P < 0.05.

In order to investigate the protective role of RES in LPS-induced ALI, the mice were challenged with LPS for 24 h, then lung histological changes were evaluated in mice with or without RES oral pretreatment for seven days. As shown in Figure 2a, the mice without RES pretreatment showed significant histological deterioration according to the lung HE stained section, such as alveolar wall thickness, edema, and inflammatory cell infiltration, which were greatly reversed by 100 mg/kg of RES-pretreated. No obvious cytotoxicity has been found in mice receiving 100 mg/kg of RES alone group (Figure 2a and b). Meanwhile, 25 mg/kg of RES had no protective effects against ALI, since lung of the mice that received 25 mg/kg of RES treatment had no significant difference compared with no treatment ALI mice (Figure 2a and b). However, lung of mice that received 50 mg/kg of RES treatment had a slight but not a significant recovery than that of no treatment or 25 mg/kg of RES treatment mice, lung of mice that received 100 mg/kg of RES treatment had a significant improvement than that of 50 mg/kg treatment mice group (Figure 2a and b). In accordance, the lung injury score system demonstrated that 100 mg/kg of RES pretreatment could efficiently attenuate the severity of ALI upon exposure to LPS for 24 h compared with ALI mice without RES pretreatment (Figure 2b). Thus, mice which received 100 mg/kg of RES treatment were used for further study. Furthermore, as a lung vascular permeability indicator, the lung W/D weight ratio significantly decreased in RES-pretreated mice compared with untreated mice, indicating that RES led to less lung edema (Figure 2c). Next, BALF cells and protein concentration were collected and determined as an index of integrity of epithelial basement membrane. Result showed that BALF cells and protein level statistically decreased in RES-pretreated mice compared with ALI mice alone, suggesting that RES had an inhibitory capacity of preventing cells and protein leakage (Figure 2d and e). Finally, in LPS-challenged mice, MPO activity in lung tissue was also prominently reduced with RES pretreatment, whereas mice without RES pretreatment had markedly raised MPO activity (Figure 2f). Taken together, RES could attenuate LPS-induced ALI.

### 3.2 RES modulated inflammatory responses in LPS-induced ALI

**Figure 3:**
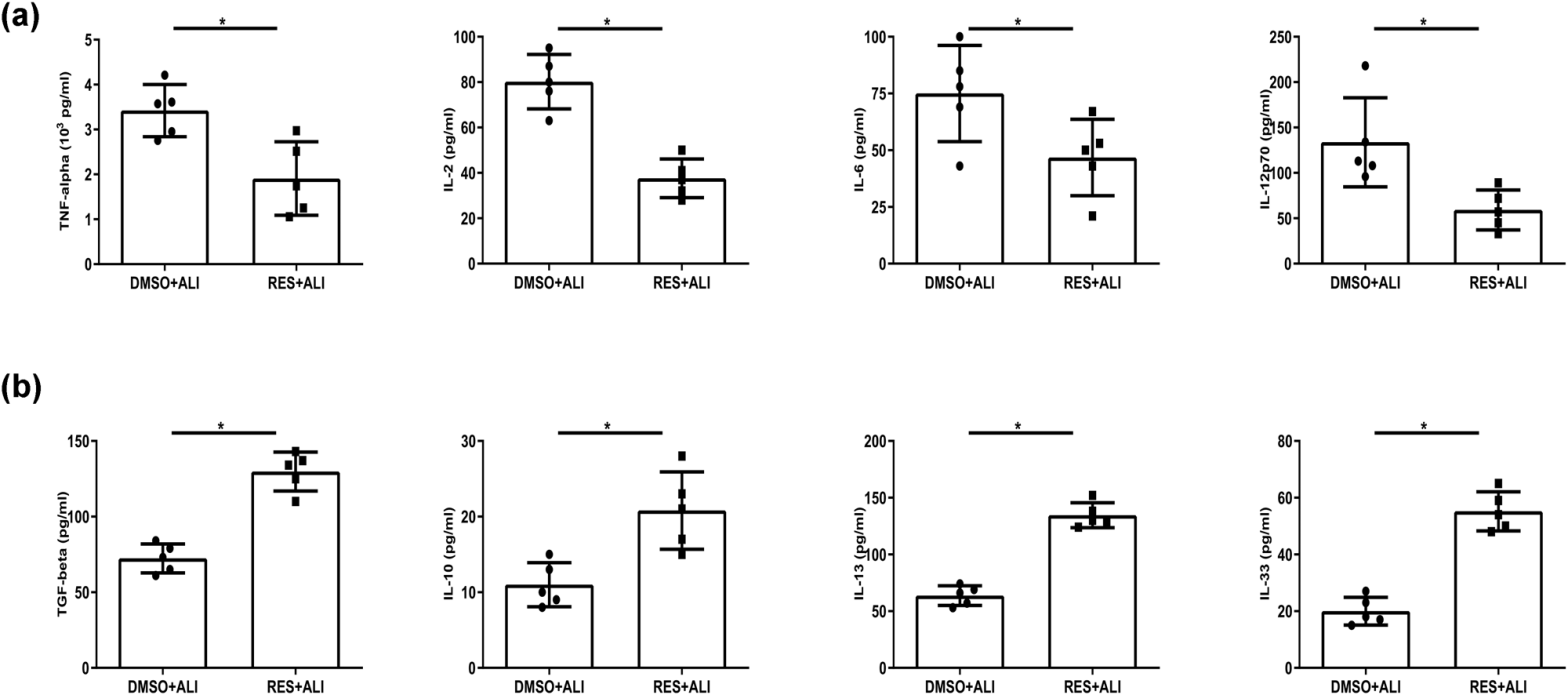
The profiles of decreased proinflammatory (Th1)-related cytokines but increased anti-inflammatory (Th2)-related cytokines 24 h after LPS challenged by RES pretreatment. The mice BALF sample were collected within 3 treatment groups at indicated time. Th1- and Th2- related cytokines were measured by ELISA kits. (a) Decreased Th1-related cytokines including TNF-α, IL-2, IL-6, and IL-12p70 were observed. (b) Increased Th2-related cytokines containing TGF-β, IL-10, IL-13, and IL-33 were determined. Data were from one representative experiment of three performed and presented as the mean value ± SEM (*n* = 5), *t*-test was used, *P < 0.05.

To address the effect of RES on pulmonary inflammation in LPS-induced ALI, the pro/anti-inflammatory cytokines in BALF were determined 24 h post LPS challenge. The level of proinflammatory cytokines, including TNF-α, IL-2, IL-6, and IL-12p70, were strikingly decreased (Figure 3a), whereas the level of anti-inflammatory cytokines, including TGF-β, IL-10, IL-13, and IL-33 were notably increased (Figure 3b) in RES-pretreated mice compared with ALI group. These data suggested that RES inhibited Th1, but promoted Th2 immune responses in LPS-induced ALI.

### 3.3 RES regulated pulmonary cDC maturation and function in LPS-induced ALI mice

**Figure 4:**
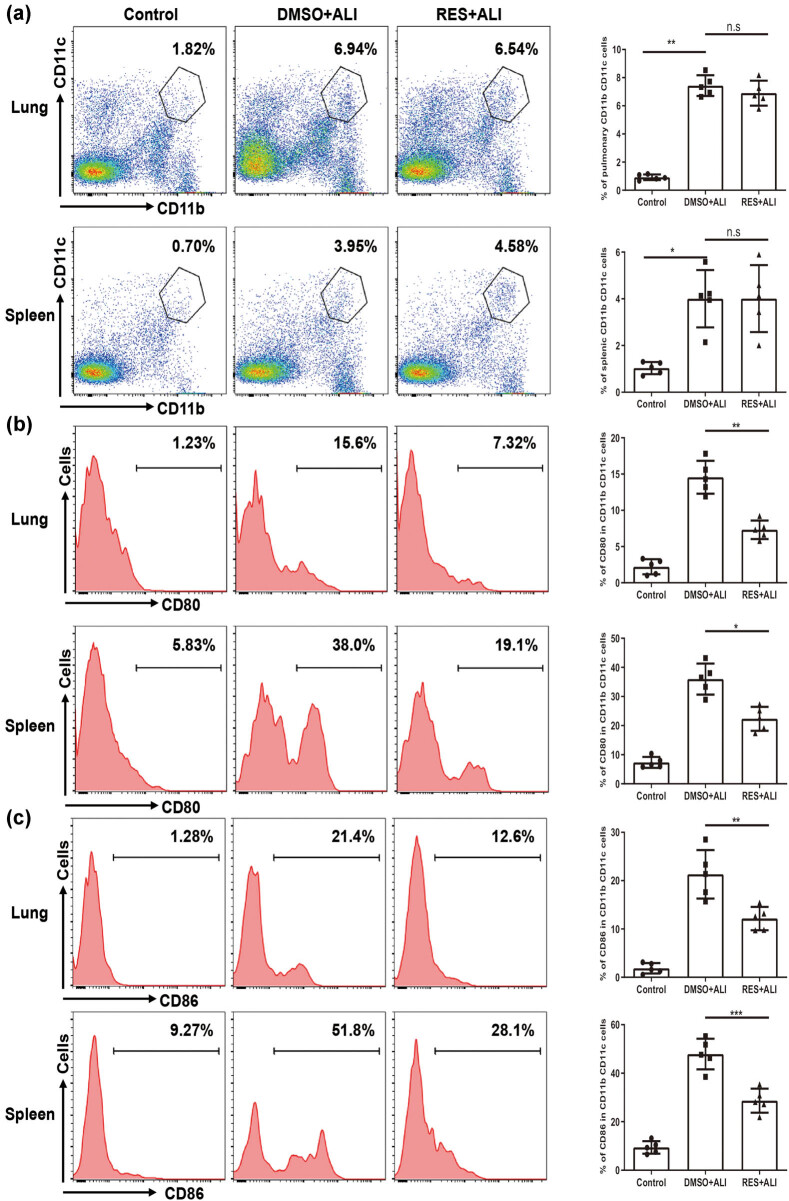

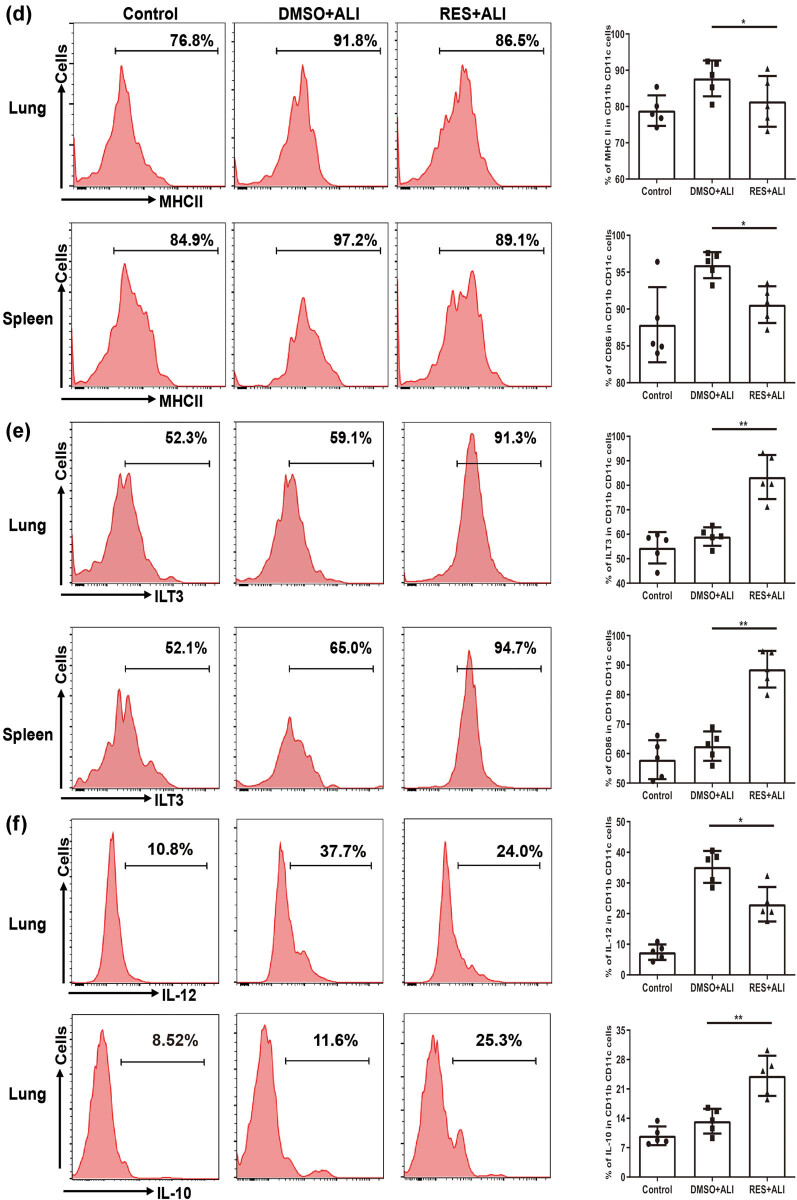
Pretreated RES has no influence on cDCs expansion, but regulated pulmonary and splenic cDCs maturation and function. 24 h after LPS exposure, cDCs’ responses were evaluated in differential treatment mice groups. (a) The representative flow cytometry profile and frequency of cDCs (CD11b^+^CD11c^+^) response in lung and spleen upon LPS challenge. Plots were pregated on CD45 cells. (b–d) The representative flow cytometry profile and frequency of co-stimulatory molecules (CD80 and CD86) and MHC II in lung and spleen upon LPS challenge. (e) The representative flow cytometry profile and frequency of inhibitory molecule ILT3 in lung and spleen upon LPS challenge. (f) The representative flow cytometry profile and frequency of IL-12 and IL-10 secretion in lung upon LPS challenge. Plots were pregated on cDCs cells (from b to f). Data were from one representative experiment of three performed and presented as the mean value ± SEM (*n* = 5), 1-way ANOVA was used, *P < 0.05, **P < 0.01, and ***P < 0.001, n.s means no significance.

Study has shown that pulmonary cDC was significantly increased in the pathological process of ALI [14]. Hence, we studied the effects of RES on cDCs in our model. First, we tested whether RES pretreatment could arrest pulmonary cDC expansion in LPS-induced ALI. Single cell suspension from lung and spleen were prepared for analysis at 24 h post LPS challenge, since splenic and pulmonary cDCs rapidly increased and maintained for 24 h [19]. Although significant pulmonary cDC expansion was observed in LPS-challenged mice compared with control mice, RES pretreatment led to slightly decreased pulmonary cDCs compared with that of non-pretreated ALI mice (Figure 4a), indicating that RES pretreatment had no significant impact on pulmonary cDC expansion. In line with pulmonary cDCs, no change in the percentage of splenic cDCs occurred (Figure 4a) 24 h after LPS exposure.

Several reports have demonstrated that the maturation of pulmonary cDCs controlled the pathogenesis of LPS-induced ALI [13,19]. Moreover, RES could influence monocytes to DCs differentiation, maturation, and function *in vitro* [35]. Meanwhile, pulmonary cDC expansion was unaffected by RES pretreatment, hence we next explored whether the maturation and function status of cDCs could be modulated by RES pretreatment in LPS-exposed mice. The co-stimulatory molecules CD80, CD86 and MHC II expression level were associated with maturation status [36]. Accordingly, we checked these molecules’ expression on pulmonary and splenic cDCs. Interestingly, the co-stimulatory molecules CD80 and CD86, and MHC II had higher expressions on both the pulmonary and splenic cDCs in LPS-challenged mice than those in control mice (Figure 4b–d). Importantly, RES pretreatment dramatically suppressed these molecules expression on both the pulmonary and splenic cDCs upon LPS challenge (Figure 4b–d). In addition, RES pretreatment led to increased ILT3 expression on both the pulmonary and splenic cDCs (Figure 4e), which was known to be a DC tolerogenic marker with a potency to induce regulatory T cells (Treg) and to regulate the balance Th cells’ immune responses [37]. Consistent with the BALF inflammatory level in Figure 3, RES-pretreated pulmonary cDCs secreted less IL-12, but more IL-10 than those of ALI mice (Figure 4f). Summarily, these data indicated that RES pretreatment might have no influence on cDC expansion, while it could regulate pulmonary cDCs maturation and function in LPS-induced ALI mice.

### 3.4 RES regulated the maturation and function of BMDCs *in vitro*

**Figure 5:**
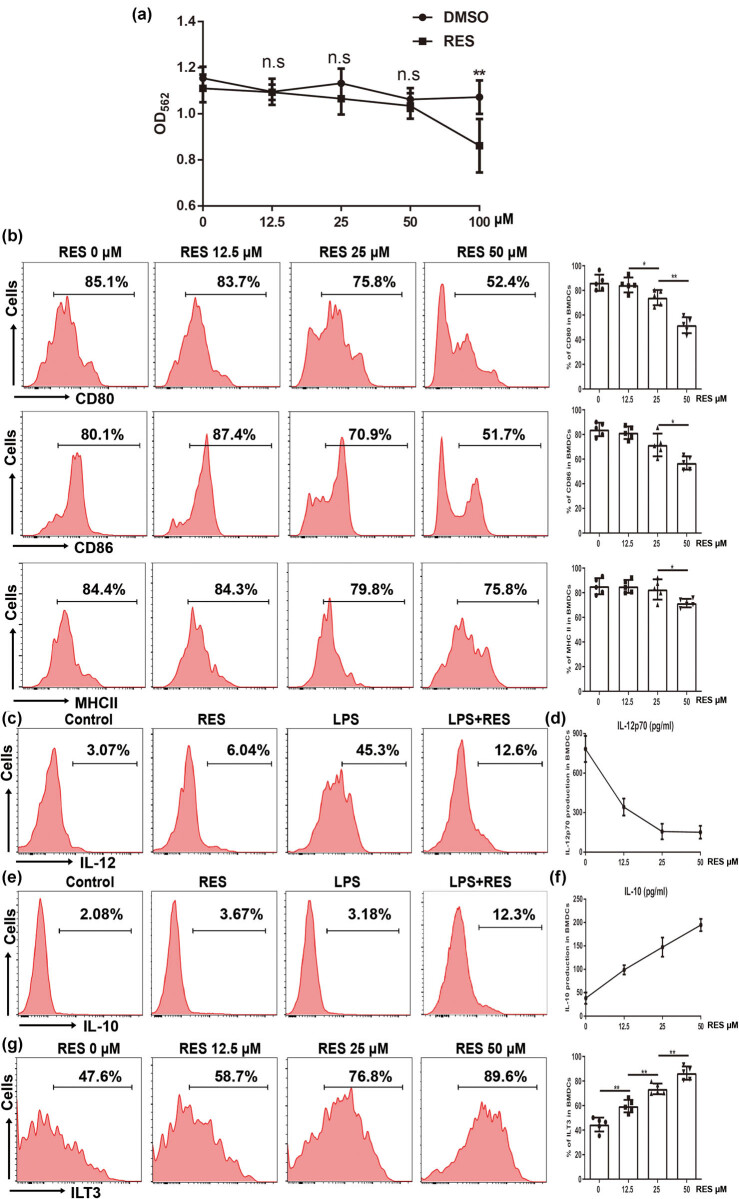
Regulation of cultured BMDCs maturation and function by RES. RES was added into BM cells on initial day to regulate BMDCs maturation combined with 40 ng/mL rmGM-CSF and 40 ng/mL rmIL-4 for 6 days, then 500 ng/mL LPS was administrated for 48 h to induce maturation. (a) The proliferation and cytotoxicity of RES on BMDCs were determined by MTT. Differential concentrations of RES wereused (0, 12.5, 25, 50, and 100 μM). (b) The representative flow cytometry profile and frequency of costimulatory molecules (CD80 and CD86) and MHC II on cultured BMDCs with various concentrations of RES treatment. (c and e) The representative flow cytometry profiles of IL-12^+^ and IL-10^+^ cells on cultured BMDCs upon LPS stimulation with RES treatment (50 μM); (d and f) the supernatant of cultured BMDCs upon LPS stimulation with various concentrations of RES treatment was determined by ELISA. (g) The representative flow cytometry profile and frequency of ILT3 on cultured BMDCs with various concentrations of RES treatment. Data were from one representative experiment of three performed and presented as the mean value ± SEM (n = 5), 1-way ANOVA was used, *P < 0.05 and **P < 0.01, n.s means no significance.

Due to the maturation and function of pulmonary cDC modulated by pretreated RES *in vivo*, we subsequently explored whether RES treatment could control the maturation and function of BMDCs *in vitro*. BMDCs were cultured and their responses to RES treatment *in vitro* were tested. First, we measured the effects of RES on BMDC viability and cytotoxicity by MTT. Various concentrations of RES were added into cultured BMDCs medium on the initial day and for eight days. Data showed that the frequency of cultured BMDCs slightly decreased compared with the untreated BMDCs *in vitro* by the administration of relatively low doses of RES ( ≤ 50 μM) (Figure 5a). However, at high dose of RES (100 μM), obvious cytotoxicity was detected so that further experiments were performed with RES at the concentration of ≤50 μM. Accordingly, RES (50 μM) did not simply obstruct the proliferation of DCs *in vitro*, in line with our *in vivo* and previous data [38]. Thus, we checked the maturation markers’ expression, which significantly decreased *in vivo*, on BMDCs on day eight by flow cytometry. As expected, CD80, CD86, and MHC II dramatically decreased in RES-treated BMDCs compared with untreated cells (Figure 5b). Subsequently, the modulation of Th1 and Th2-skewed cytokines by RES on BMDCs was confirmed by IL-12 and IL-10 expressions and secretion, since lower IL-12^+^ cells frequency (Figure 5c) and lower supernatant of IL-12 (Figure 5d), but higher IL-10^+^ cells frequency (Figure 5e) and higher supernatant of IL-10 (Figure 5f) were found by cultured BMDCs in the presence of RES at various concentrations for eight days than those of control counterpart. Interestingly, the tolerogenic marker ILT3, which increased in the ALI mice model, also increased in a dose-dependent manner *in vitro* (Figure 5g). Taken together, these data revealed that the maturation and function of BMDCs could also be regulated by RES treatment *in vitro*.

### 3.5 BMDCs by RES pretreatment regulated CD4 T cells producing more IL-10, but less IL-17 *in vitro*

**Figure 6:**
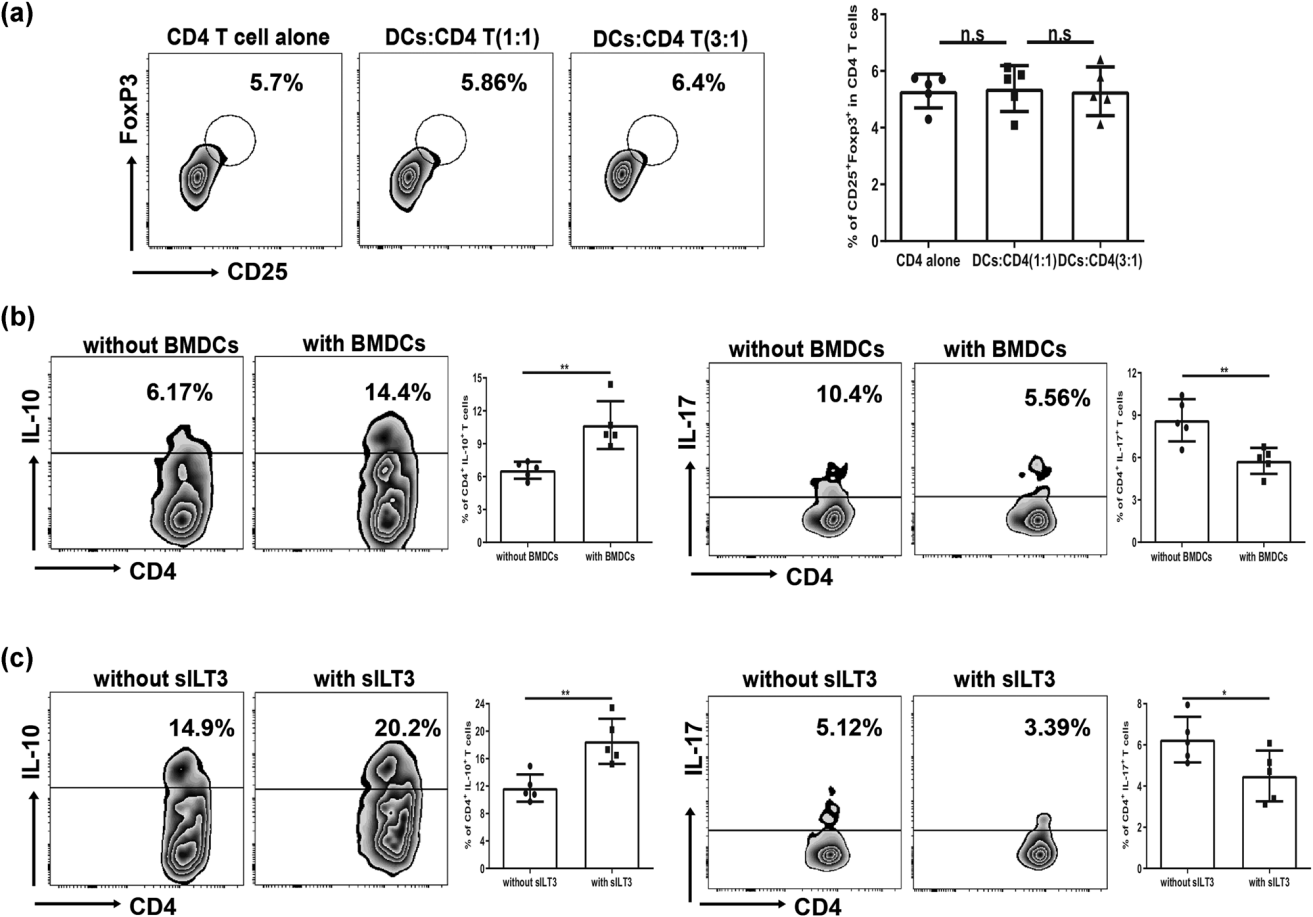
Regulation of CD4 T cells-driven IL-10 and IL-17 secretion by RES treated BMDCs. RES (50 μM) treated BMDCs were co-cultured with sorted CD4 T cells from ALI mice with rmIL-2 (5 ng/mL), rhTGF-β (5 ng/mL), and rmIL-6 (20 ng/mL) for three days. (a) The representative flow cytometry profile and frequency of CD25^+^Foxp3^+^ cells in co-cultured system (DCs:CD4 T = 1:1 or 3:1, respectively). Plots were pregated on CD4^+^ cells. (b) The representative flow cytometry profile and frequency of CD4^+^IL-10^+^ cells and CD4^+^IL-17^+^ cells from co-cultured system (DCs:CD4 T = 3:1). (c) The representative flow cytometry profile and frequency of CD4^+^IL-10^+^ cells and CD4^+^IL-17^+^ cells from co-cultured system (DCs:CD4 T = 3:1) in the presence of sILT3 protein (10 μg/mL). Data were from one representative experiment of three performed and presented as the mean value ± SEM (*n* = 5), *t*-test was used, *P < 0.05 and **P < 0.01, n.s means no significance.

The balance between Th1 and Th2, which are crucial to maintain the homeostasis of immune system, is one of causes for the development and progression of ALI [39]. The balance between Treg and Th17 cells has been confirmed to be involved in ALI/ARDS [40]. Our result also indicated that ILT3, which has an ability to modulate Th cell immune responses, was significantly upregulated in cDCs. Thus, to answer whether RES treatment could affect BMDCs mediated CD4 T cells toward Treg or Th17 polarization *in vitro*, we co-cultured RES-treated BMDCs with sorted splenic CD4 T cells from ALI mice for three days. Results showed that BMDCs did not affect CD4 T cells toward Treg polarization, since the increase in CD25^+^Foxp3^+^ cells on CD4 T cells was not significant (Figure 6a), but it promoted CD4 T cells secreting more IL-10 but less IL-17 compared with its untreated counterpart (Figure 6b). To our surprise, even more IL-10 but less IL-17 secretion on sorted CD4 T cells from ALI mice in the co-culture system was regulated by the administration of sILT3 protein (10 μg/mL) (Figure 6c), indicating that highly expressed ILT3 on BMDCs might mediate CD4 T cells producing IL-10 and IL-17. Hence, these results showed that BMDCs by RES treatment might have no impact on CD4 T cells toward Treg polarization, but could mediate more IL-10 and less IL-17 production on CD4 T cells.

### 3.6 RES-treated BMDCs enhanced the recovery of ALI

**Figure 7:**
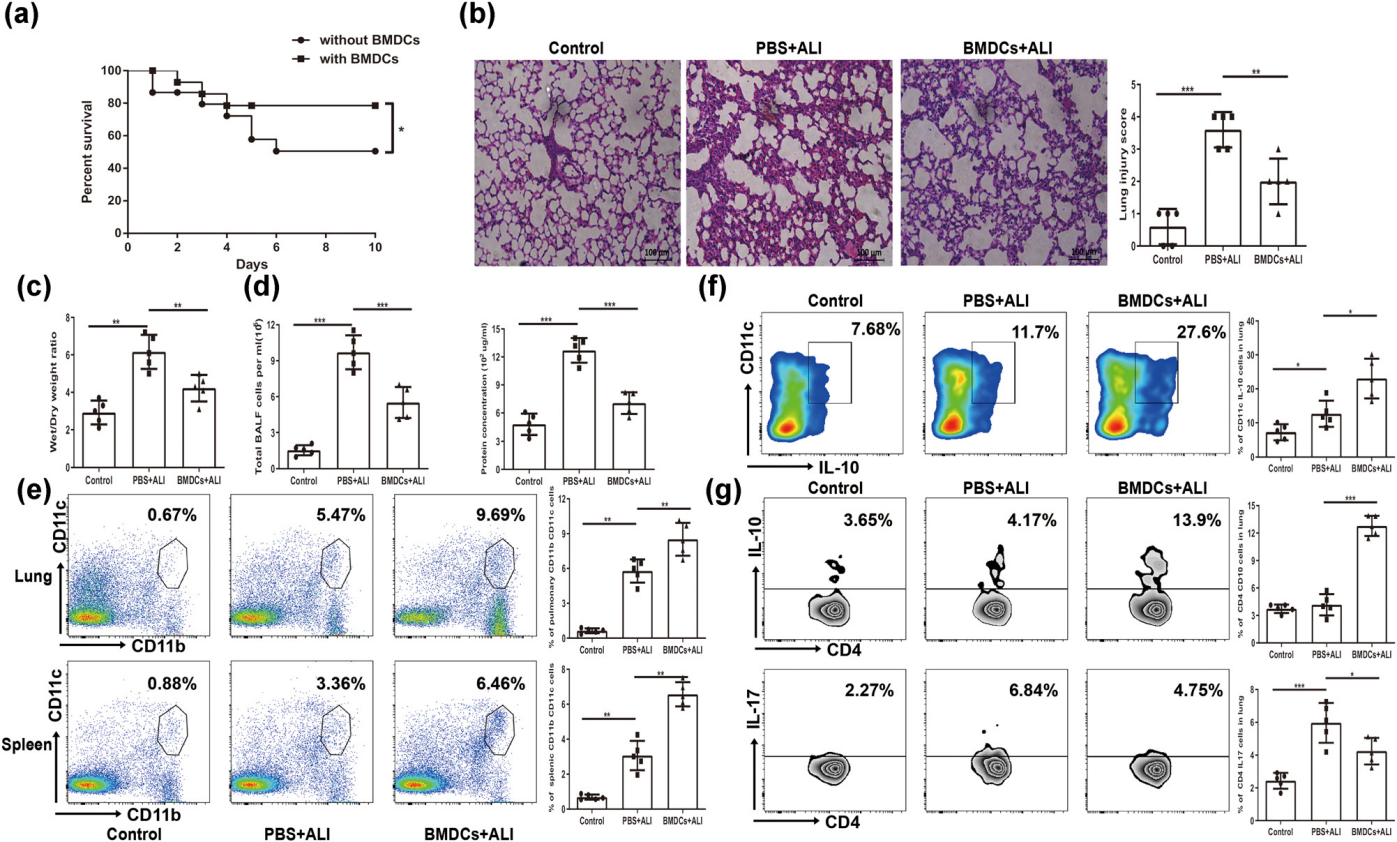
Adoptive transfer RES treated BMDCs attenuates ALI. 1 × 10^6^ RES treated BMDCs *i.v* one day before LPS-challenged ALI mice. (a) The survival rate of mice was observed until day 10 following LPS challenge. (b) Pulmonary histopathological scores were determined at 24 h after LPS challenge (magnification: 200×). (c) The wet lung-to-dry weight ratio (W/D ratio) was monitored at 24 h after LPS challenge. (e) Total cells and proteins in BALF were analyzed at 24 h after LPS challenge. (e) The representative flow cytometry profile and frequency of CD11b^+^CD11c^+^ cells in lung and spleen from ALI mice. (f) The representative flow cytometry profile and frequency of pulmonary CD11c^+^IL-10^+^ cells from ALI mice. (g) The representative flow cytometry profile and frequency of peripheral CD4^+^IL-10^+^ and CD4^+^IL17^+^ cells from ALI mice. Data were from one representative experiment of three performed and presented as the mean value ± SEM (*n* = 5), 1-way ANOVA was used, for survival assay, *n* = 15, Kaplan–Meyer analysis with log-rank test was used, *P < 0.05, **P < 0.01, and ***P < 0.001.

To determine whether RES-treated BMDCs had a potential protective role in ALI mice, C57BL/6 mice were adoptively transferred with 1 × 10^6^ RES-treated BMDCs through the tail vein one day before LPS challenge. We first observed the survival rate of ALI mice that received RES-treated BMDCs. Data showed that the survival rate (80%) of mice in tolerogenic BMDCs received group was higher than that (54%) of the unreceived group (Figure 7a). We then used a histological evaluation of lungs to confirm the effect of RES-treated BMDCs in ALI mice. Lung section showed a severity of alveolar wall thickness and inflammatory cell infiltration without treatment at 24 h post LPS challenge, whereas the histological damage of lungs of mice which received RES-treated BMDCs were moderated (Figure 7b). W/D ratio and BALF cell and protein levels in BMDC-transferred mice were improved compared with those of untreated mice (Figure 7c and d).

Mechanistically, compared with untreated mice, the BMDC-transferred mice showed a higher proportion of cDCs in lung and spleen (Figure 7e), which produced more IL-10 (Figure 7f). Additionally, the mice that received BMDCs had a great increase in the proportion of IL-10 producing CD4 T cells, but a sharp decrease in the proportion of IL-17 producing CD4 T cells in the lung compared with untreated mice (Figure 7g). These data indicated that BMDCs by RES treatment could improve the recovery of ALI.

## 4 Discussion

As a globally high mortality and morbidity disease, ARDS, the severe type of ALI, is often described as the acute onset of diffuse bilateral pulmonary infiltrates by chest radiograph with PaO_2_/FiO_2_ ≤ 300 mmHg or no clinical evidence of left atrial hypertension [41]. The central issue of the development of ARDS/ALI is generally accepted to be uncontrolled inflammation after the beginning of description of ARDS in 1967 [42], leading to the devastation of the pulmonary epithelium and the disruption of the blood–air barrier. Besides pulmonary uncontrolled inflammation, oxidative stress during the injury process, which supply an excess amount of molecular oxygen to the tissues [43,44], also play an important role in ALI [26]. However, over the past decades, although great achievement has been made in the field of understanding the pathogenesis of ARDS/ALI, such as inositol could improve the surfactant functions and reduce IL-6 levels [45] or targeting IL-6 and other cytokine-induced “cytokine storm” [46] to alleviate ARDS, little convincing therapeutic strategies or agents are available to fully control the excessive inflammation until now. Thus, finding novel treatment approaches to govern the superfluous inflammation seems to be critical.

LPS-induced ALI in mice, which have similarities in features with clinical sepsis-driven ALI in humans, could be used for research on therapeutic approaches to control inflammation. In the present study, our findings demonstrated that LPS-induced ALI at the early stage led to a rapid cDC accumulation in mice (Figure 4a). RES, a natural polyphenol produced by plants, could partially mitigate ALI in mice (Figure 2). In contrast to previously reported mechanisms, we found that partial protection against lung injury in ALI mice by RES might result from the regulation of the maturation and function of cDC *in vivo* (Figures 4 and 7), possibly through regulating influence the balance between proinflammation and anti-inflammation in ALI mice (Figure 3). Moreover, RES could also control the maturation and function of BMDCs *in vitro*, which, in accordance with our *in vivo* data, downregulated CD80, CD86, and MHC II expressions, while upregulating the expression of the tolerance marker ILT3 in a dose-dependent manner (Figure 5). Additionally, RES-treated DCs induced CD4 T cell producing more IL-10, but less IL-17 *in vitro* (Figure 6). Finally, BMDCs by RES treatment adoptively transferred into ALI mice accelerated protection against lung injury in LPS-driven ALI mice (Figure 7).

In humans and mice, DCs can be divided into two subsets: cDCs and pDCs. They are crucial to the priming phase of immune responses [8]. Lung, as a mucosal organ, is widely distributed with DCs, where the majority is immunocompetent cDCs, while the minority is immunoregulatory pDCs [47]. Moreover, no ligands (TLR2 and TLR4) for LPS exist on the pDCs’ surface, hence, pDC maturation does not respond to LPS [48]. Therefore, in the current work, we primarily centered on pulmonary cDCs. Indeed, previous evidences have demonstrated that respiratory cDCs extensively participated in a large amount of lung inflammation diseases, such as allergy, respiratory viral infection, and inflammation-mediated lung injury [19–21]. Consistent with previous reports, at the early stage of ALI (24 h) [36], the aggregation/expansion status of pulmonary and splenic cDCs was observed in ALI mice (Figure 4a). This finding indicated that cDC aggregation/expansion might induce the Th1 immune responses. In fact, Figure 2 showed that Th1-related cytokines in BALF had been statistically elicited by LPS exposure in ALI mice. As an essential secondary signal, CD80/CD86 links with its receptors on immune cells to DCs, act as messengers between innate and adaptive immune systems. The main function of MHC II on DCs is to present the processed short peptides to activate adaptive immune responses. Thereby, the expression level of co-stimulatory molecules on DCs surface such as CD80 and CD86 and MHC II reflects the degree of DC maturation and function. Our results showed that the maturation of pulmonary and splenic cDCs led to higher expressions of CD80, CD86, and MHC II molecules and large amount of IL-12 secretion upon LPS challenge in mice (Figure 4b–d and f). This result indicated that matured DCs played a direct role in the pathogenesis of lung injury in ALI. Additionally, besides the direct influence on ALI, cDCs might also indirectly contribute to the pathogenesis of lung injury since *in vitro* BMDCs co-cultured with sorted CD4 T cells from ALI mice resulted in more IL-17 secretion compared with RES-treated groups (Figure 6b). Accordingly, more CD4^+^IL-17^+^ cells were found in ALI mice (Figure 7g).

RES, a well-known polyphenolic compound in plants, has a great anti-inflammatory property, inhibiting oxidative stress [49] or NLRP3 inflammasome [26] to prevent ALI. However, the role of RES in the pathology of inflammation in ALI is not well-elucidated. Published and our present data showed that RES could suppress DC maturation and induce its tolerance in humans and mice [31,35]. Therefore, the repression of DC expansion and maturation with RES might offer a way to attenuate lung injury upon LPS challenge. In line with our hypothesis, daily oral administration of RES for seven days prior to LPS challenge strikingly improved the pulmonary pathology deterioration, decreased the pulmonary edema, reduced the protein leakage, and increased the MPO activity (Figure 2). Meanwhile, the pathology of inflammation was also alleviated, manifesting decreased proinflammatory cytokines, but increased anti-inflammation cytokines after RES pretreatment (Figure 3). Interestingly, the regulation of cDC maturation and function rather than aggregation/expansion was witnessed by RES pretreatment in ALI mice, as reflected by the declining of CD80, CD86, and MHC II (Figure 4a–d). Although RES pretreatment could decrease CD80, CD86 and MHC II on DCs, it did not neglect that these membrane antigen expression changes in DCs might depend on the presence of lung inflammatory circumstances such as increased mediators and cytokines. Indeed, reports have showed that cytokines (e.g., TNF-α) could alter membrane antigen expression *in vitro* [50]. Meanwhile, the obstruction of cDC aggregation/expansion was indeed observed, and the concentration of the RES utilized was too high (100 μM) to apply in practice *in vitro* (Figure 5a). To our surprise, ILT3, an important DC tolerance marker, significantly increased after RES pretreatment *in vivo* and *in vitro* (Figure 4e and 5e), indicating that regulated cDCs by RES pretreatment might have a capacity to induce repression of immune responses.

Regarded as “professional” APCs, DCs are believed to be critical determinants of Th cell polarization. Indeed, the severity of lung injury in ALI was associated with imbalanced T cell subsets [6]. Among T cell subsets, the balance between Th17 and Treg is an important risk indicator of early ARDS/ALI [51]. The balance toward Th17 polarization might accelerate the immuno-pathogenesis of ALI [52], whereas the balance toward Treg polarization might contribute to the recovery from inflammation and lung repairs of ALI [53]. Hence, we next investigated the ability of cDCs in the polarization of CD4 T cells of ALI mice after RES pretreatment. Although Figure 6a suggested that the RES pretreated cDCs failed to induce the CD4 T cells polarize toward Tregs, CD4 T cells produced IL-10 (Figure 6b), reveals that the protection of ALI by cDCs might not be on account of the shifting CD4 T cell toward Treg cell polarization, but owing to the inhibitory effect of IL-10 secretion. In agreement with our presumption, IL-17 expression was remarkably decreased by cDCs co-cultured with CD4 T cells from ALI mice (Figure 6b), suggesting the halt of Th17 polarization induced by cDCs in our setting. Interestingly, the regulation of CD4 T cells secreting more IL-10, but less IL-17 might depend on ILT3 expression on cDC surface, indicating that ILT3 protein could amplify the phenotype of CD4 T cell producing more IL-10 but reduced IL-17 production (Figure 6c).

As a novel approach, DC-based immune therapy is extensively used in cancer, infection, and other diseases [54–56]. Our data pointed out that RES pretreatment could regulate the maturation and function of cDCs *in vitro* and *in vivo*. Hence, we assessed the therapeutic role of cDCs by RES pretreatment in protection against ALI in mice. Surprisingly, adoptive transfer of RES pretreated DCs greatly promoted the survival of ALI mice (Figure 7a). Meanwhile, the severity of lung injury in ALI was also partially ameliorated (Figure 7b).

In conclusion, our study showed that RES pretreatment could efficiently mitigate the severity of lung injury in ALI, and that RES participated in protecting against the pathological process of ALI by modulating the maturation and function of cDCs. Our results might further extend our understanding of a protective role of RES against ARDS/ALI.

## Figures and Tables

**Figure A1:**
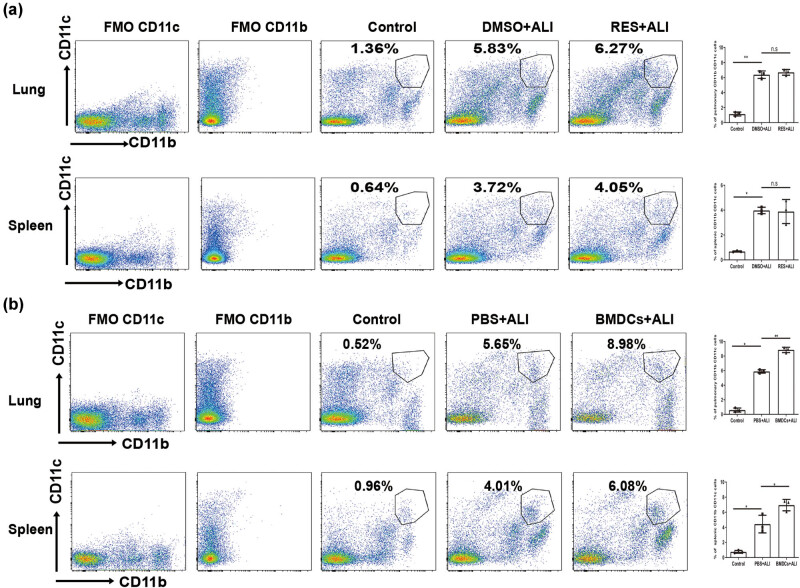
FMO control to define CD11b^+^ CD11c^+^ cells. (a) The representative flow cytometry profile of cDCs (CD11b^+^ CD11c^+^) with FMO control in lung and spleen upon LPS challenge (RES pretreatment). (b) The representative flow cytometry profile of CD11b^+^ CD11c^+^ cells with a FMO control in lung and spleen from ALI mice (adoptive transfer of BMDCs).

**Figure A2:**
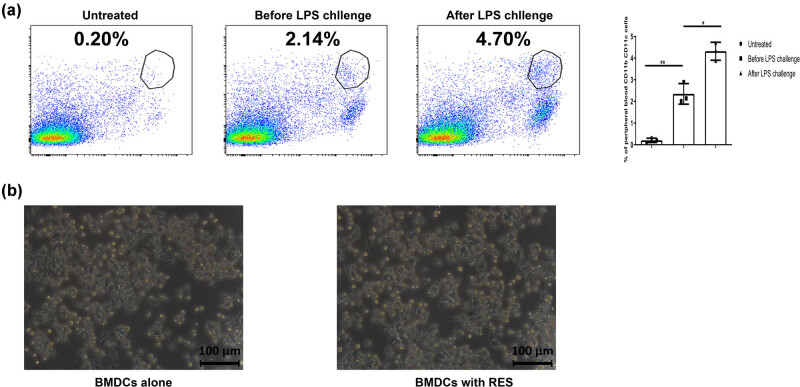
BMDCs in peripheral blood before and after LPS challenge and the morphology between untreated BMDCs and RES-treated BMDCs. (a) The representative flow cytometry profile of cDCs (CD11b^+^ CD11c^+^) in the peripheral blood. (b) Morphology of *in vitro* cultured BMDCs were shown.
